# An unfolding monkeypox outbreak in Europe and beyond

**DOI:** 10.1186/s40779-022-00394-z

**Published:** 2022-06-15

**Authors:** Gabriel Luz Wallau, Rafael Maciel-de-Freitas, Jonas Schmidt-Chanasit

**Affiliations:** 1 Departamento de Entomologia and Núcleo de Bioinformática, Instituto Aggeu Magalhães (IAM), Fundação Oswaldo Cruz (Fiocruz), Avenida Fernando Simões Barbosa, s/n, Cidade Universitária, Recife, Brazil; 2 Department of Arbovirology, Bernhard Nocht Institute for Tropical Medicine, WHO Collaborating Center for Arbovirus and Hemorrhagic Fever Reference and Research, National Reference Center for Tropical Infectious Diseases, Bernhard-Nocht-Straße 74, 20359 Hamburg, Germany; 3 Laboratório de Mosquitos Transmissores de Hematozoários, Instituto Oswaldo Cruz, Fundação Oswaldo Cruz (Fiocruz), Avenida Brasil, 4365, Manguinhos, Rio de Janeiro, Brazil; 4 Faculty of Mathematics, Informatics and Natural Sciences, University of Hamburg, Ohnhorststrasse 18, 22609 Hamburg, Germany

**Keywords:** Monkeypox virus, Poxvirus, Zoonotic virus, Human transmission

Dear Editor,

Monkeypox is a zoonotic disease caused by the monkeypox virus (MPXV), a double-stranded DNA virus in the genus *Orthopoxvirus*, family Poxviridae. MPXV is known to be transmitted between animals such as rats and squirrels, but animal-to-human and human-to-human transmission has been known in Africa since 1970. Transmission is linked to direct contact with body fluids, skin lesions, and patient items as well as respiratory droplets in case of prolonged face-to-face contact [1]. Until recently, only a few cases were associated with travel to endemic countries or contact with infected animals imported from endemic countries. However, in non-endemic countries, outbreaks showed limited onward community transmission. The time from exposure to onset of symptoms ranges from 5 to 21 d. It has been suggested that infections in adults may cause mostly mild symptoms, but in young children, pregnant women and immunocompromised persons, severe symptoms may also occur which can be fatal in 3–10% of cases [2]. Typical symptoms such as fever, swollen lymph nodes, rash, and skin lesions are more common in patients’ face, hands, and feet but can also appear on the genital and in the eyes.

Poxviruses have a relatively lower point mutation rate compared to single-stranded RNA viruses, but are more prone to other genome-wide genetic changes such as recombination, gene loss and gain. Two clades are known to infect animals including humans: The West African (WA) and Central Africa (Congo Basin clade—CB) clades [3, 4], with limited data suggesting differences in terms of pathogenicity and transmission. Strains belonging to the CB clade have shown higher pathogenicity and transmission efficiency between humans.

After hundreds of human MPXV cases without travel history to endemic countries were reported in non-endemic countries in 2022, the MPXV outbreak attracted substantial concern from scientists and health authorities. From May 13th to June 2nd, there were 780 MPX cases confirmed worldwide including 688 laboratory confirmed cases in EU/EEA member states. Cases have been predominantly detected so far in Spain (156) and Portugal (138) followed by Germany, France and Netherlands with 57, 33 and 31 diagnosed patients, respectively [5]. The detection of only WA clade strains, which is expected to be less efficiently transmitted between humans, and the apparently higher incidence of cases in men who have sex with men (MSM) raise several questions about the MPXV transmission mode and evolution. For instance, has the MPXV-WA lineage acquired the capacity for more efficient human-to-human transmission? Does MPXV change tissue tropism or infection dynamics allowing a more efficient sexual transmission? Does community transmission in non-endemic countries start from a single or from multiple introduction events? Research groups have successfully sequenced several MPXV genomes and evidence suggests that a single introduction of the WA clade occurred in Europe with further spreading events that disseminated this specific strain to other countries [6]. In addition, the MPXV-WA lineage strains associated with the recent outbreak differ by about 40 nucleotide mutations from MPXV strains sequenced 4 years ago, suggesting that the evolutionary rate may have increased from one mutation per genome per year to 12 mutations per genome per year [6].

In order to promote a global joint response effort, an incisive leadership of the World Health Organization (WHO) is expected, for instance, by providing guidelines to strengthen countries’ preparedness and to better inform the MSM community, as well as by improving sequencing and bioinformatic support needed for MPXV genome analysis associated with rapid data sharing to facilitate molecular epidemiology and investigation of basic research questions. The continuous release of sequenced MPXV genomes daily will allow the assessment of the introduction and transmission routes into and among countries and evaluation of the impact of genomic changes on viral fitness changes, if there are any. Data shared so far point to a much less dramatic situation in comparison to the severe acute respiratory syndrome coronavirus 2 (SARS-CoV-2) pandemic. The high proportion of symptomatic patients plus the easy-to-identify signs of monkeypox infection promote rapid and effective identification of infected patients. Thus, contact tracing, patient interviews, and increased smallpox vaccination for those in contact with infected patients are critical to map and control further transmission. Moreover, current evidence suggests that the contagious phase of MPXV coincides with the symptomatic phase which allows rapid detection and isolation of affected individuals curtailing new transmission events [2]. Lastly, because MPXV is a virus on the high priority list of potential human threat as defined by the WHO because of its close relationship with smallpox and its known infection capacity in humans, there are several medical treatment options including antiviral compounds and vaccines. The recent MPXV outbreak is one example in a long list of increasing spillover events that impact human health. Spillover of zoonotic viruses is clearly associated with human encroachment to the remaining pristine habitats, intensive raising of livestock animals and continued interaction with animals overall. The MPXV is another example of a pathogen with a large and complex network of poorly known reservoir species which makes new spillover events difficult to predict and control. On the other hand, there are tools available, such as genomic surveillance, to timely study and control outbreaks. There is an urgent need to apply these new methodologies within the one health concept. Thus, we will be able to create risk maps of urban-animal interfaces and strengthen our pandemic preparedness. The swift response to outbreaks is the “new normal for humankind” and we should use all tools available to control and minimize the impact of emerging and reemerging human pathogens.

## Data Availability

All information and data were extracted from published material quoted in the reference section.

## Abbreviations

MPXV: Monkeypox virus
MSM: Men who have sex with men
SARS-CoV-2: Severe acute respiratory syndrome coronavirus 2
WHO: World Health Organization
